# Distinguishing *Astragalus mongholicus* and Its Planting Soil Samples from Different Regions by ICP-AES

**DOI:** 10.3390/molecules21040482

**Published:** 2016-04-12

**Authors:** Lin Li, Sihao Zheng, Qingzhen Yang, Shilin Chen, Linfang Huang

**Affiliations:** 1 Institute of Medicinal Plant Development, Chinese Academy of Medical Sciences & Peking Union Medical College, Beijing 100193, China; 13323258786@163.com (L.L.); zshzha@163.com (S.Z.); 2 Department of Pharmacy, The First Hospital of Fangshan District, Beijing 102400, China; 15281083341@163.com; 3 Institute of Chinese Materia Medica, China Academy of Chinese Medical Sciences, Beijing 100700, China; slchen@implad.ac.cn

**Keywords:** *Astragalus membranaceus*, inorganic elements, *daodi* herb, soil, ICP-AES

## Abstract

“*Daodi* herb” enjoys a good reputation for its quality and clinical effects. As one of the most popular *daodi* herbs, *Astragalus membranaceus* (Fisch.) Bge var. *mongholicus* (Bge.) Hsiao (*A. membranaceus*) is popularly used for its anti-oxidant, anti-inflammatory and immune-enhancing properties. In this study, we used inductively coupled plasma atomic emission spectrometry (ICP-AES) technique to investigate the inorganic elements contents in *A. mongholicu* and its soil samples from *daodi* area (Shanxi) and non-*daodi* areas (Inner Mongolia and Gansu). A total of 21 inorganic elements (Pb, Cd, As, Hg, Cu, P, K, Zn, Mn, Ca, Mg, Fe, Se, B, Al, Na, Cr, Ni, Ba, Ti and Sr) were simultaneously determined. Principal component analysis (PCA) was performed to differentiate *A. mongholicu* and soil samples from the three main producing areas. It was found that the inorganic element characteristics as well as the uptake and accumulation behavior of the three kinds of samples were significantly different. The high contents of Fe, B, Al, Na, Cr and Ni could be used as a standard in the elements fingerprint to identify *daodi* and non-*daodi* *A. Mongholicus.* As the main effective compounds were closely related to the pharmacodynamics activities, the inter-relationships between selected elements and components could reflect that the quality of *A. Mongholicus* from Shanxi were superior to others to a certain degree. This finding highlighted the usefulness of ICP-AES elemental analysis and evidenced that the inorganic element profile can be employed to evaluate the genuineness of *A. mongholicus*.

## 1. Introduction

The term “*daodi* herb” refers to a concept that has been widely recognized in Chinese medicinal history for centuries. It is defined as “medicinal material that is produced, cultivated, harvested and processed in specific geographic regions with designated natural conditions, ecological environment and particular attention”. These factors lead to quality and clinical effects surpass those of the same botanical origin produced from other regions, and thus is widely recognized and has long enjoyed a good reputation. “*Daodi* herb“ is also known as authentic and superior medicinal herbal, geo-herb, authentic medicinal, geo-authentic medicinal material and genuine medicinal material [1].

The dried root of *Astragalus membranaceus* (Fisch.) Bge var. *mongholicus* (Bge.) Hsiao (*A. membranaceus*), one of the most popular *daodi* herbs, has been widely used as a traditional prescription medicine more than 2000 years in China [2]. Due to its anti-oxidant, anti-inflammatory and immune-enhancing properties, *A. mongholicus* has gained popularity and been used extensively as a dietary supplement to improve health and prevent diseases, such as inflammation, heart disease, aging, and cancer [3,4]. *A. mongholicus* is mainly grown in the northeast, north, and northwest of China as well as in Mongolia and Korea (*A. membranaceus*). Shanxi is the *daodi*-producing areas of *A. mongholicus*, whose products are famous for “high quality, good effect and large amount”.

*A. mongholicus* is recorded in European Pharmacopoeia (EP, 8.0 version), British Pharmacopoeia (BP, 2013 version), Japanese Pharmacopoeia (JP, 16 version) and Chinese Pharmacopoeia (CP, 2015 version). American “Dietary Supplement Health and Education Act” (DSHEA), European Food Safety Authority (EFSA) and Ministry of Health China (MOH) also recorded *A. mongholicus* as an herbal dietary supplement. As a dietary supplement, *A. mongholicus* has abundant biologically active ingredients; organic and inorganic compounds including carbohydrates, proteins; elements like Ca, P, K, Fe, and Mg; vitamins (A, C, and B-complex); and dietary fibers [3]. With so many inorganic elements, *A. Mongholicu*s can be a source of minerals with important physiological functions in the human organism. Ca is a major component of bone and response for increasing the density of the capillary wall, and reducing its permeability and leakage [5]. Zn is a co-factor in enzymes, some of which are structures of the nervous system [6]. Being part of hemoglobin, Fe is involved in the transportation of oxygen [7]. K and Na play an indispensable role in fluid osmotic pressure and acid-base balance [8]. Abundant inorganic elements in herbs are the formation of some active chemical constituents, thus influencing the medical properties [9]. It is well-known that environmental factors such as soil, climate, light, and humidity directly influence the secondary metabolites (many of which are bioactive constituents) of plants. Studies have shown that mineral nutrition can affect the metabolism of many bioactive constituents in herbs, such as flavonoids, saponin and so on [10,11]. It was reported that the flavonoids, polysaccharide and saponin in *Ophiopogon japonicus* have a direct relationship with K, Fe, Mn, B, Ba and Zn in its planting soil [12]. The optimum concentration of K significantly increased the yields of rosmarinic acid, ursolic acid, oleanolic acid and flavonoids in *Prunella vulgaris* L. [13]. In *Salidroside*, salidroside was negatively correlated with P and K [14]. At present, the inorganic elements have become a vital factor to evaluate the quality of Chinese herbal medicine originating from different regions and also help in tracking and judging its authenticity according to certain inorganic elements characteristic. Therefore, determination of the type and content of inorganic elements in *daodi* medicinal materials is essential for understanding their efficacy.

Methods for the determination of inorganic elements are flame atomic absorption spectrometry (FAAS) [15], inductively coupled plasma mass spectrometry (ICP-MS) [16], atomic fluorescence spectrometry (AFS) [17], and so on. Nowadays, the use of ICP-AES is becoming more common in analyzing numerous inorganic elements and obtaining fingerprints of the element pattern. In this way, the technique has been successfully applied to fruits [18], pharmaceutical materials [19], eggs [20] and flowers [21]. As for *A. Mongholicus*, previous papers were mainly focused on the discrimination of different species and limited studies focus on its origin areas, which was not sufficient and comprehensive enough to research the geoherbalism of the famous traditional Chinese medicine. Our previous studies have shown that the contents of chemical compositions in *A. mongholicus* from Shanxi were greater than those in Inner Mongolia and Gansu, which suggested that the *daodi* *A. mongholicus* is better than others [22,23,24]. Are there differences in inorganic elemental characteristics in *A. mongholicus* from *daodi* and non-*daodi* areas? In this study, 21 inorganic elements (Pb, Cd, As, Hg, Cu, P, K, Zn, Mn, Ca, Mg, Fe, Se, B, Al, Na, Cr, Ni, Ba, Ti and Sr) in *A. mongholicus* and soil samples were determined by ICP-AES. The differences between *daodi* and non-*daodi* *A. mongholicus* were then investigated, thereby providing a theoretical basis for the relationship between the elements and the medicinal value of Chinese herbal medicine.

## 2. Results and Discussion

### 2.1. Internal Quality Controls of ICP-AES Procedure

#### 2.1.1. Detecting Wavelength and Detection Limit

The determination was investigated for each element by recording the spectra of the sample solution and potentially interfering elements near the analytical line. First, Select the spectral lines for detection that have little spectral interference and high precision. A compromise between the most sensitive spectral line of each analyte and lower background interference was used to select the optimum spectral lines for this study. The capability of the method as a routine analysis method was estimated through the determination of the limits of detection (LOD, mg/L) and limits of quantification (LOQ) of every element studied. The LOD was calculated according to Boumans using 3σ and LOQ using 9σ for pure element standards by measuring an appropriate reagent blank solution ten times and digested sample solutions three times. The elemental analysis wavelengths and the LOD values are presented in Table 1. The LOD values for the 21 elements were in the range of 0.01–0.48 mg/L. Compared with the determined results of samples, the LOD values were adequate for almost all elements to determine the elements in preserved samples.

#### 2.1.2. Calibration Studies

To determine the linearity of the response *vs.* concentration for the elements, a series of standard solutions for ICP-AES were analyzed. The calibration curves and regression analysis on calibration curves are presented in Table 1. A good linear relationship between the corresponding sensitivities and the concentrations of the elements was achieved. All linear correlation coefficients were greater than 0.9990.

#### 2.1.3. Accuracy and Precision of the Method

To ensure the accuracy of the experiment, spiking experiments of each sample were conducted. Each standard solution whose concentration was close to sample was added into the sample. Samples with added after standard digestions were determined using ICP-AES. Results from the recovery experiments for all elements in herb samples after digestion are listed in Table 1. For acceptance of data, the average recovery rates had to fall within a range of 85.0%–114.0%, which demonstrated that the method has acceptable accuracy and is suitable for simultaneous determination of 21 inorganic elements in *A. mongholicus* and its soil samples. The precision expressed as relative standard deviation (RSD) ranged from 0.5% to 8.1%.

### 2.2. Inorganic Elements in the Soil

**Figure 1:**
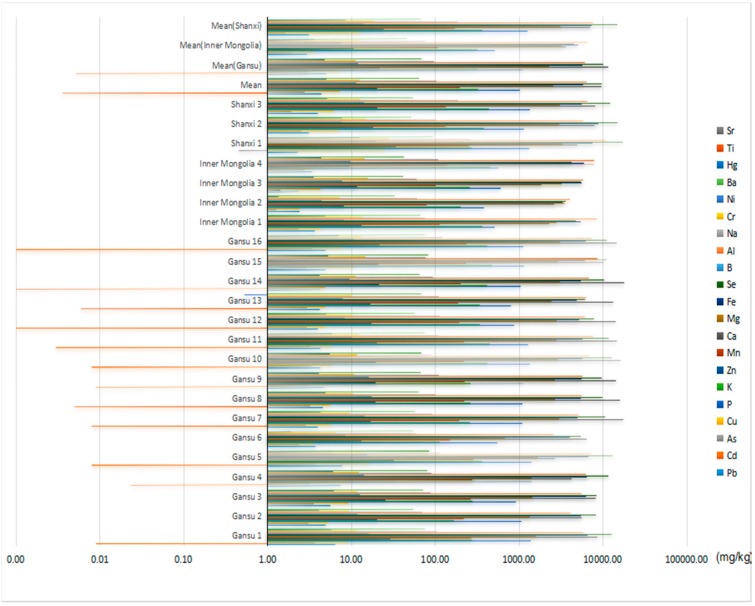
Elemental concentrations in soil samples.

From the 21 studied elements, 18 elements (Pb, Cd, As, Cu, P, K, Zn, Mn, Ca, Mg, Fe, Se, B, Al, Na, Cr, Ni and Ba) were found in soil samples obtained from Shanxi, Inner Mongolia and Gansu. For the rest of elements, the contents found in all samples were lower than the limit of detection (LOD) values. The results are shown in Figure 1. By comparing the total contents of the 18 elements in the soil samples, we found that the contents of some elements varied largely. For example, the average content of Se in soil samples from Shanxi was 14,890 mg/kg while that from Inner Mongolia was 4539 mg/kg. The highest amounts of P found in samples of Shanxi was 1338 mg/kg, whereas the lowest level from Inner Mongolia was 386 mg/kg. In the case of Ca, the mean concentration of samples from Gansu was almost four-fold higher than that from Inner Mongolia. However, the difference for some elements was not so marked. For example, the mean concentration of K was 321 mg/kg in the soil samples from Inner Mongolia and 324 mg/kg in that from Gansu. Furthermore, the inorganic elements in all soil samples were seen to decrease in the following order: Se > Ca > Al > Fe > Mg > P > K > Mn > Na > Ba > Zn > Cr > B > Cu > Ni > Pb > As > Cd.

According to the theory of biogeochemistry, the accumulation of inorganic elements in plants has direct relationships with the geochemical distribution and migration of the element, the chemical composition of geological and metallogenic backgrounds and parent material of the soil, namely, plants cultivated in different geological environments probably vary in the accumulation of inorganic elements, thus vary in pharmacological effects. Only herb cultivated in those special soils may meet the standard of *daodi* traditional Chinese medicine.

### 2.3. PCA for Inorganic Elements in Soil of A. mongholicus

**Figure 2:**
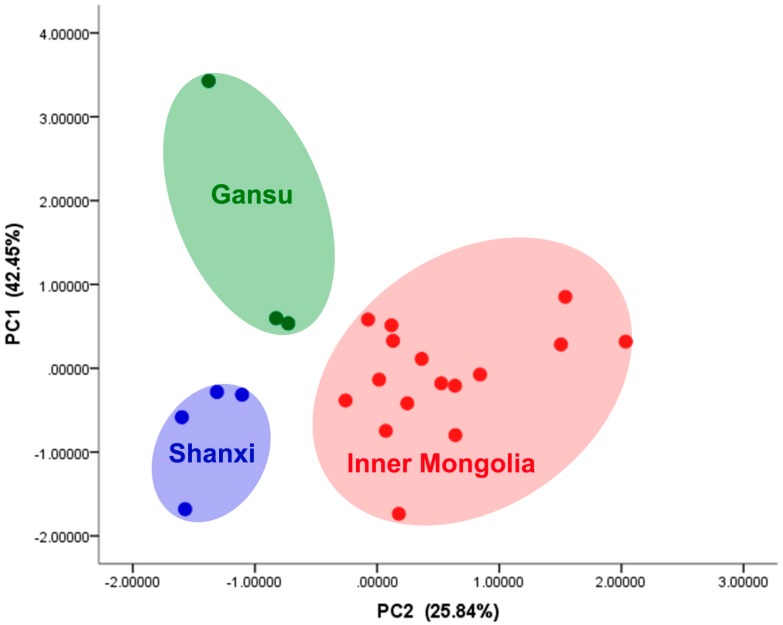
PCA for inorganic elements in soil.

Principal component analysis (PCA) is a method to describe the relationships of multidimensional data arrays between variables and objects by greatly reducing data and presenting different manners for interpretation. Thus far, PCA has emerged as a promising tool for the classification of food samples in terms of its type, level, provenance, and quality. There were three groups in the scores plot (Figure 2), composed of soil samples from Shanxi, Inner Mongolia and Gansu, respectively, indicating differences in their mineral composition in soil. Soil samples from different producing areas could by completely separated from each other without overlap, which showed that inorganic elements could distinguish soil samples from different producing areas effectively.

### 2.4. Contents of Inorganic Elements in A. mongholicus

**Figure 3:**
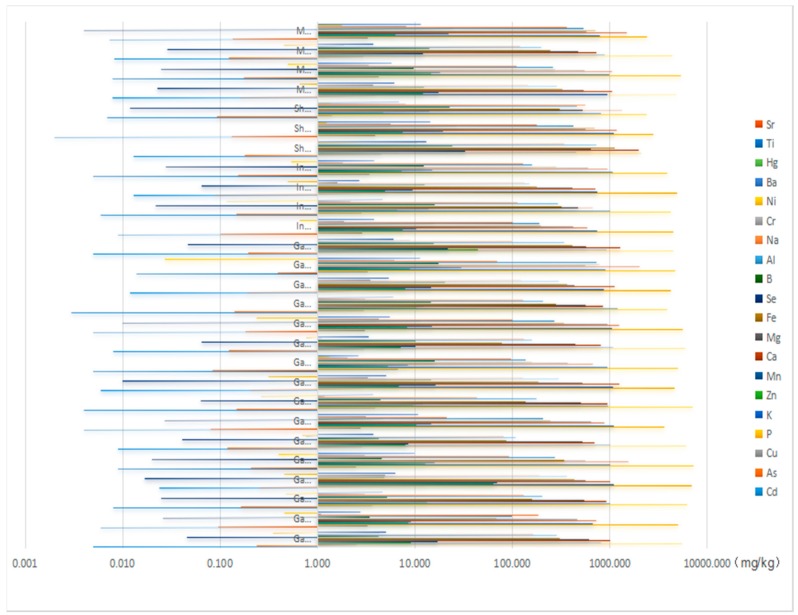
Elemental concentrations in herb samples.

All of the elemental concentrations obtained in *A. mongholicus* samples from the three sampling sites are summarized in Figure 3. As shown, the contents of the 18 elements ranged from 0 to 7292 mg/kg; thus, the differences of the contents between various elements were significant. The P was, quantitatively, the most abundant mineral, having a mean concentration of 4859 mg/kg. Other elements, such as Ca, K, Mg and Fe, were also presented in large amounts in the samples with mean concentrations of 1068 mg/kg, 949 mg/kg, 546 mg/kg and 328 mg/kg, respectively. In all analyzed plant samples, the contents of Se and Cd did not exceed 0.1 mg/kg. According to the different geographic regions analyzed, it can be concluded that Shanxi samples contained almost 2~4 fold the levels of Fe, B, Al, Na, Cr, Ni and Ba than samples from Inner Mongolia and Gansu. Among them, Fe, Cr and Ni are necessary trace elements for human body according to the World Health Organization (WHO) [25]. In contrast, the Shanxi samples were poorer in P, K and Se than other samples, especially Se was 6~7 fold levels lower. Moreover, Gansu included much higher content of Zn in comparison with other origins. For the rest of the elements, the three regions had no significant variability.

Every *daodi* herb may have its own characteristic elements fingerprint. Our results showed that the difference in contents of inorganic elements among the three origins was significant. The high contents of Fe, B, Al, Na, Cr and Ni may be used as a standard in the elements fingerprint to identify *daodi* and non*-daodi* *A. mongholicus* [26].

### 2.5. PCA for Inorganic Elements in A. mongholicus

**Figure 4:**
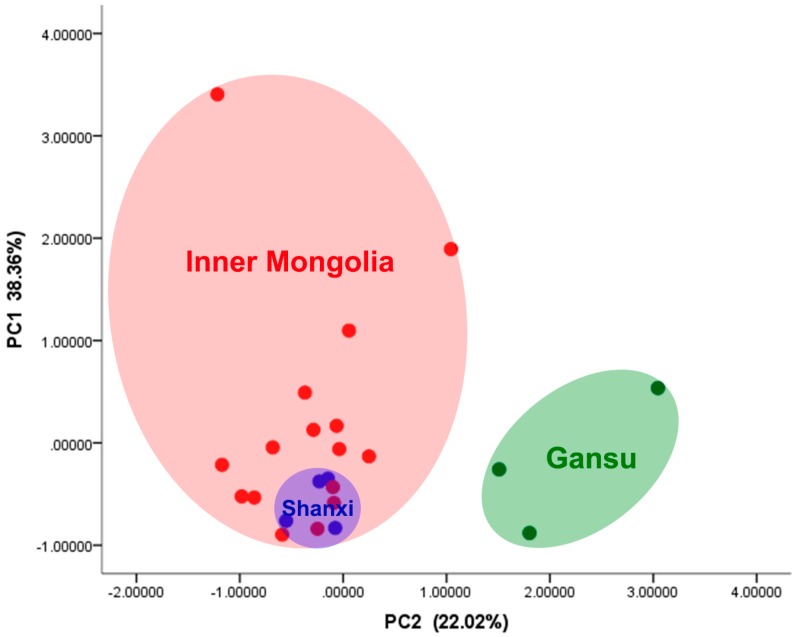
PCA for inorganic elements in herbs.

According to the scores plot (Figure 4), the formation of two main groups was observed. One group was formed exclusively by the herb samples from Shanxi, and the other group was perceptibly formed by two subgroups, namely Gansu and Inner Mongolia. This separation was attributed to the closeness of the sampling points. The result showed that the *A. mongholicus* from Gansu was significantly different from the other origins and the Shanxi and Inner Mongolia samples had some similarities in mineral composition by gathering in the left part. The application of PCA confirmed the differences between *daodi* and non*-daodi* *A. mongholicus*.

### 2.6. Uptake and Accumulation Behavior

**Figure 5:**
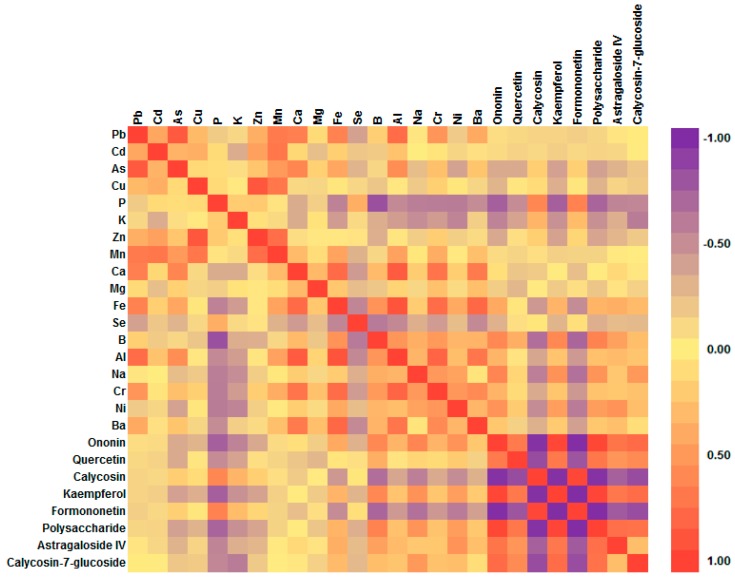
Correlation analysis between the contents of inorganic elements and effective components of *A. mongholicus*.

Given that each plant has its own inorganic elements uptake and accumulation behavior, the content in plant depending on content in soil was not considered justifiable [27]. The correlation data in terms of linear correlation coefficient values between inorganic elements and chemical compositions that were significant at 95% and 99% confidence level was examined. The results are shown in Figure 5. Ca and Fe showed high and significant correlations at 99% confidence level with Al, Cr and Ba. Mn also showed this tendency with Pb, Cd and Cu, whereas K and P had high negative correlations with most of the inorganic elements. The results indicated that the uptake of these elements in *A. mongholicus* might have a synergistic or inhibiting effect.

**Figure 6:**
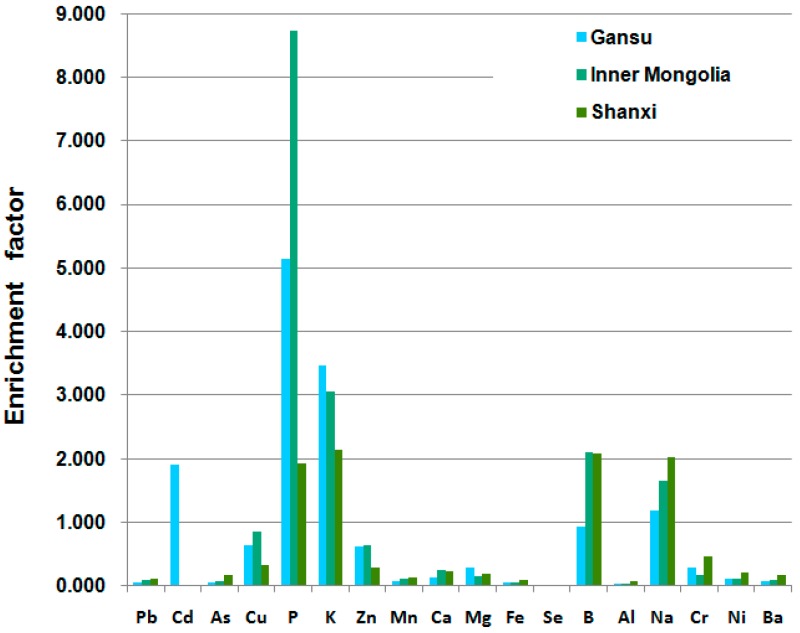
The enrichment factors of inorganic elements in *A. mongholicus*.

Enrichment factor is applied to measure the uptake and accumulation behavior of plants. Once the value is more than 1, the plant accumulates inorganic elements from soil [28]. Figure 6 shows the enrichment factor of all *A. mongholicus* samples. The results showed that *A. mongholicus* had a high ability of enrichment for P, K, Na and B with the enrichment factor of 5.334, 3.184, 1.412 and 1.336, respectively. Samples collected from Shanxi accumulated considerably more Fe, Al, Na, Ni, Cr and As than the others, whereas the Cd, Cu, P, K and Zn in contrast is 2~4 times lower. Moreover, *A. mongholicus* from Gansu had a moderate enrichment capacity. Plants from different geographical origins exhibited their own accumulation behavior, which is the basis for herb characterization by elemental pattern and can be applied for *daodi* and non-*daodi* *A. mongholicus* identification.

### 2.7. Correlation Analysis

Our previous study has shown the correlation between ecotype and quality of *A. mongholicus* [22,23,24]. The inter-relationships between selected elements and components in *A. mongholicus* were explored by correlation analysis. Figure 5 showed that P, K and Zn had similar significant negative correlations with most of the chemical compositions. As the *daodi* region, plant samples from Shanxi were poor in P, K and Zn. Further more, the majority of the inorganic elements except P were negatively correlated with calycosin and formononetin. The other inorganic elements and chemical compositions also had inhibition or synergy interaction with each other in different degrees. As the main effective compounds were closely related to the pharmacodynamics activities, the contents of selected elements could reflect that the quality of *A. Mongholicus* from Shanxi were superior to others to a certain degree.

## 3. Materials and Methods

### 3.1. Reagents and Apparatus

The experimental water was ultra pure water prepared by Milli-Q (18.2 MΩ, Millipore, Bedford, MA, USA) water system. Concentrated HNO_3_ and HCl used for digestion were of analytical grade and were obtained from Beijing Chemical Works. All glassware was cleaned with nitric acid prior to use. Mixed standard solution was prepared by diluting 1000 mg/L standard solution of every different elements. A graphite furnace (EHD36-LABTECH, LabTech, Beijing, China) was employed to digest the samples. The analytes were determined by ICP-AES (ICAP6300, Thermo Scientific, West Palm Beach, FL, USA) in a simultaneous mode. Working parameters of ICP-AES are shown in Table 2.

### 3.2. Sample Preparation

Materials were collected in October 2012, including herbs and soil. The soil was scraped from the roots of herbs. The herbs were identified as *Astragalus membranaceus* (Fisch.) Bge var. *mongholicus* (Bge.) Hsiao by Professor Lin-Fang Huang associated with a researcher who came from the Key Laboratory of Bioactive Substances and Resources Utilization of Chinese Herbal Medicine, Ministry of Education, Institute of Medicinal Plant Development, Chinese Academy of Medical Sciences & Peking Union Medical College. All herbs were washed thoroughly with tap water followed by distilled water, dried at 105 °C, grounded with a mortar and stored in the plastic bags. The soil samples were dried at 105 °C, grounded with a mortar and stored in the plastic bags.

About 1 g of the herbal samples was accurately weighed into a quartz digestion vessel. One milliliter of concentrated HNO3 and 3 mL of concentrated HCl were added to the vessel. The mixture was placed on a graphite furnace for about 12 h at 60 °C before the sample was digested. After complete digestion and removal of acid for 2 h at 120 °C, the samples were transferred into a volumetric flask and made up to 25 mL with ultrapure water. The solution was taken over through the 0.22 μm membrane filter and then determined by ICP-AES. The soil samples were processed in the same way.

### 3.3. Calibration Procedure

A multi element standard (1000 mg/L) was used for the preparation of standard solutions in 2% HNO_3_ as well as in matrix-matched solutions for the digest solutions. The multi element stock solution was diluted to the following ten standard concentrations (mg/L): 0.05, 0.10, 0.20, 0.50, 1.0, 2.0, 5.0, 10, 15, and 20. Blank solutions were prepared in the same way. Calibration ranges were modified according to the expected concentration ranges of the elements.

### 3.4. Spiking Procedures

Spiking of *A. mongholicus* samples and soil samples were carried out by addition of aqueous multi element standard solutions to a set of samples prepared as described above. All spiked samples were prepared in triplicate and measured by ICP-AES.

## 4. Conclusions

In the present study, an inductively coupled plasma atomic emission spectrometry method was firstly developed for investigating inorganic element characteristics of *A. mongholicus* and its planting soil from different regions. With this approach, 21 inorganic elements were identified. Data in this work evidenced that the inorganic element characteristics as well as the uptake and accumulation behavior were significantly different among *A. Mongholicus* from different producing areas. The high contents of Fe, B, Al, Na, Cr and Ni could be used as indicators in the elements fingerprint to identify *daodi* and non-*daodi* *A. Mongholicus.* Moreover, as the main effective compounds were closely related to the pharmacodynamics activities, the contents of selected elements may reflect that the quality of *A. Mongholicus* from Shanxi is superior to others to some extent. This finding was in good agreement with our previous conclusions that the contents of effective components in *A. mongholicus* from Shanxi were more than those from Inner Mongolia and Gansu. The research provided scientific data for further study of the relationship between the content of elements and its medical therapy, and furthermore, offered references for the quality evaluation and geo-herbalism assessment of *A. mongholicus* from the perspective of inorganic elements, which will facilitate its clinical application.

## Figures and Tables

**Table 1:** Work curve, limit of detection and recovery.

Element	Wavelength (nm)	Regression Equation	LOD, mg/L	LOQ, mg/L	Average Recovery (%)	RSD (%)
Pb	220.54	y = 171.5763x + 2.0716	0.24	0.72	109.7	3.5
Cd	228.50	y = 2712.5644x + 6.1782	0.04	0.12	93.7	5.5
As	193.69	y = 159.8310x − 1.5560	0.01	0.03	108.7	6.5
Cu	324.75	y = 4410.2400x − 4.4859	0.07	0.21	95.3	5.8
P	213.62	y = 16.8398x − 0.0549	0.11	0.33	96.7	1.6
K	766.49	y = 3246.1758x − 114.7232	0.08	0.24	113.3	3.3
Zn	213.85	y = 2226.9064 + 6.2482	0.08	0.24	104.3	2.9
Mn	257.61	y = 14,069.62x + 4.9874	0.03	0.06	91.7	5.6
Ca	317.93	y = 5090.8325x + 15.5628	0.14	0.42	89.0	4.5
Mg	279.50	y = 100,774.53x + 14.3562	0.17	0.51	88.3	3.5
Fe	259.93	y = 2422.3150x − 0.5618	0.06	0.18	100.3	8.1
Se	196.02	y = 92.5530x + 0.9983	0.20	0.60	100.7	3.0
B	249.67	y = 3074.1732x + 12.5746	0.02	0.06	93.3	3.3
Al	308.21	y = 1544.3306x − 17.5037	0.04	0.12	106.7	0.5
Na	589.59	y = 1605.8634x − 313.4292	0.09	0.27	93.3	1.6
Cr	267.71	y = 5825.6772x + 8.5930	0.08	0.24	85.0	4.7
Ni	231.60	y = 1401.1639x − 0.0311	0.06	0.18	113.7	4.2
Ba	455.40	y = 61,217.94x + 202.9208	0.48	1.44	111.0	2.4
Hg	194.16	y = 475.7408x + 2.5548	0.06	0.18	98.0	6.4
Ti	334.94	y = 1892.2594x + 15.4562	0.09	0.27	94.0	3.8
Sr	407.77	y = 3059.3525x + 5.526	0.11	0.33	114.0	4.0

**Table 2:** The operating parameters of instrument.

Parameters	Value
Output power	1.2 kw
Auxiliary air flow	0.2 L/min
Atomization gas flow	0.8 L/min
Cooling flow	16 L/min
The observing pattern	Bidirection
The flux of the elevation solution	1.5 mL/min
RF-Generator	40 MHz
Replicates for each analysis run	3
Sample uptake delay	30 s
Viewing mode	Axial
Spray chamber type	Cyclonic
Sample propulsion	Peristaltic pump, three channel
Torch type	Fassel type
Detector	Segmented-array charge-coupled (SCD)

## References

[1] Zhao Z., Guo P., Brand E. (2012). The formation of daodi medicinal materials. J. Ethnopharmacol..

[2] Chinese Pharmacopoeia Commission (2010). Pharmacopoeia of the People’s Republic of China.

[3] Hong M.J., Ko E.B., Park S.K., Chang M.S. (2013). Inhibitory effect of *Astragalus membranaceus* root on matrix metalloproteinase-1 collagenase expression and procollagen destruction in ultraviolet B-irradiated human dermal fibroblasts by suppressing nuclear factor kappa-B activity. J. Pharm. Pharmacol..

[4] Ma C.H., Wang R.R., Tian R.R. (2009). Calycosin 7-*O*-β-d-glucopyranoside, an anti-HIV agent from the roots of Astragalus membranaceus var. mongholicus. Chem. Nat. Compd..

[5] Hepler P.K. (2005). Calcium: A central regulator of plant growth and development. Plant Cell.

[6] Khanif Y.M., Saleem M. (2013). Role of zinc in plant nutrition—A review. Am. J. Exp. Agric..

[7] Olaiya C.O., Oyewole O.E. (2010). The importance of mineral elements for humans, domestic animals and plants: A review. Afr. J. Food Sci..

[8] Zheng Q., Shen Q., Guo S. (2013). The critical role of potassium in plant stress response. Int. J. Mol. Sci..

[9] Abou-Arab A.A.K., Donia M.A.A. (2000). Heavy metals in Egyptian spices and medicinal plants and the effect of processing on their levels. J. Agric. Food Chem..

[10] Liu D.H., Guo L.P., Huang L.Q., Jin H. (2010). Effects of mineral nutrition on metabolism of flavonoids in medicinal plants. Chin. J. Chin. Mater. Med..

[11] Zeng Y., Guo L.P., Yang G., Chen B.D., Wang J.Y., Huang L.Q. (2012). Effect of environmental ecological factors on Saponins of medicinal plant. Chin. J. Exp. Tradit. Med. Form..

[12] Zhang L., Ye Z., Guo Q. (2010). Effects of soil factor on active components of Radix Ophiopogonis. Chin. Mater. Med..

[13] Chen Y.H., Yu M.M., Zhu Z.B. (2013). Optimisation of Potassium Chloride Nutrition for Proper Growth, Physiological Development and Bioactive Component Production in *Prunella vulgaris* L.. PLoS ONE.

[14] Yan X.F., Wu S.G., Wang Y. (2004). Soil nutrient factors related to salidroside production of Rhodiola sachalinensis distributed in Chang Bai Mountain. Environ. Exp. Bot..

[15] Khajeh M., Moghaddam A.R.A., Sanchooli E. (2010). Application of Doehlert Design in the Optimization of Microwave-Assisted Extraction for Determination of Zinc and Copper in Cereal Samples Using FAAS. Food Anal. Methods.

[16] Tolalioglu S. (2012). Determination of trace elements in commonly consumed medicinal herbs by ICP-MS and multivariate analysis. Food Chem..

[17] Dos Santos Walter N.L., Dannuza D.C., Samuel M.M. (2013). Slurry Sampling and HG AFS for the Determination of Total Arsenic in Rice Samples. Food Anal. Methods.

[18] Cindric I.J., Krizman I., Zeiner M., Kampic S., Medunic G. (2012). ICP-AES determination of minor- and major elements in apples after microwave assisted digestion. Food Chem..

[19] Tu Q., Wang T.B., Antonucci V. (2010). High-efficiency sample preparation with dimethylformamide for multi-element determination in pharmaceutical materials by ICP-AES. J. Pharm. Biomed..

[20] Tu Y.G., Zhao Y., Xu M.S. (2013). Simultaneous Determination of 20 Inorganic Elements in Preserved Egg Prepared with Different Metal Ions by ICP-AES. Food Anal. Methods.

[21] Toyama T. (2003). Analysis of metal elements of hydrangea sepals at various growing stages by ICP-AES. Biochem. Eng. J..

[22] Fu J., Yang S.H., Huang L.F. (2013). Simultaneous Determination of Six Flavonoid Active Components in Radix Astragali by UPLC. Chin. Pharm. J..

[23] Yang Q.Z., Liu D.W., Wang D.M. (2014). Correlation on quality and ecotype of *Astragalus membranaceus* (Fisch.) Bge. var. *mongholicus* (Bge.) Hsiao. Chin. Tradit. Herb. Drugs.

[24] Fu J., Huang L.F., Zhang H.T. (2013). Structural features of a polysaccharide from *Astragalus membranaceus* (Fisch.) Bge. var. *mongholicus* (Bge.) Hsiao. J. Asian Nat. Prod. Res..

[25] WHO/FAO/IAEA (1996). Trace Elements in Human Nutrition and Health.

[26] Yan L.N. (2008). Study on the Effective Components of *Atractylodes* Plants and *Atractylodes macrocephala* & Establishment of Herbs’ Element Fingerprint. Master’s thesis.

[27] Christine S.A., Mattusch J., Reisser W., Wennrich R. (2004). Uptake and accumulation behaviour of angiosperms irrigated with solutions of different arsenic species. Chemosphere.

[28] Kaushik A., Kansal A., Santosh, Meena, Kumari S., Kaushik C.P. (2009). Heavy metal contamination of river Yamuna, Haryana, India: Assessment by metal enrichment factor of the sediments. J. Hazard. Mater..

